# CPR related injuries of the chest wall: direct and indirect fractures

**DOI:** 10.1007/s00068-024-02678-6

**Published:** 2025-01-12

**Authors:** Anne Schenderlein, Johannes Groh, Florian Kern, Mario Perl, Stefan Schulz-Drost

**Affiliations:** 1 Department of Trauma and Orthopedic Surgery, University Hospital Erlangen, Friedrich-Alexander University Erlangen-Nürnberg (FAU), Erlangen, Germany; 2 Department of Trauma Surgery, Helios Kliniken Schwerin, Wismarsche Str. 393-397, 19055 Schwerin, Germany; 3 Department of Anaesthesiology, Friedrich-Alexander-Universität Erlangen-Nürnberg (FAU), Krankenhausstr. 12, 91054 Erlangen, Germany

**Keywords:** CPR, Rib fractures, Fracture morphology, Indirect fractures, Chest wall injury, Flail chest

## Abstract

**Background:**

Rib and sternum fractures are common injuries associated with cardiopulmonary resuscitation (CPR). The fracture mechanism is either direct by application of force on sternum and anterior ribs or indirect by bending through compression of the thorax. The aim of this study was to determine morphologies of rib fractures after CPR and to reevaluate prior findings on fracture localisation, type and degree of dislocation.

**Methods:**

The present study was based on all inpatients treated for chest wall fractures after non traumatic cardiac arrest at a Level 1 Trauma Centre from 2010 to 2016 who had received CT scans. Each fracture was analyzed for location, degree of dislocation and fracture type classified according to AO/OTA and CWIS. We also analysed Fracture Line orientation.

**Results:**

We enrolled 40 patients with a total of 423 rib fractures. We found most fractures anterolaterally between the 3rd to 6th rib symmetrically on both sides of the thorax. We found sternum fractures in 30% of the patients, 50% being located at the at the corpus sterni between rib 3 and 4. All patients with sternum fractures suffered from rib fractures and most had fractures of the cartilage or osteochondral junction. All cartilage fractures were straight, undisplaced type A fractures. Most indirect fractures occurred anterolaterally between 50 and 60° in the axial plane. More than 90% of those fractures were classified as type A, 70% showed a straight fracture line and 60% were undisplaced. There was no difference in degree of dislocation between straight and oblique fracture lines. We found 143 incomplete fractures.

**Conclusion:**

We confirmed prior findings regarding fracture patterns in CPR related injuries. We observed approximately 2–3 times as many straight-lined fractures as oblique ones following indirect trauma. One third of all fractures are incomplete, these highlights the special characteristics like high elasticity of ribs.

## Background

Skeletal injuries of the chest wall after cardiopulmonary resuscitation (CPR) are largely unavoidable, with the most common injuries being fractures of the 3rd through 5th ribs on both sides of the thorax and sternum fractures (SF) at a corresponding level. These fractures are well documented in terms of frequency, location, and severity; however, fracture morphology has not yet been the main focus [1–5]. Additionally, some fractures have been noted to show incomplete bone disruption [6].

Kissling and Hausmann were the first to examine rib fracture morphology using a manual testing machine on isolated human ribs [7]. They identified several morphological features of rib fractures, including fracture line orientation, types of fracture edges, and cortical fragments.

In clinical practice, computed tomography (CT) scanning is the most effective imaging modality for diagnosing rib fractures [8–10]. Our study specifically focuses on evaluating fracture line orientation, recognizing this as the only morphological feature assessable through CT scans. Kissling and Hausmann found that direct fractures predominantly had straight fracture lines, while indirect fractures displayed a balanced distribution of straight and oblique lines.

**Fig. 1:**
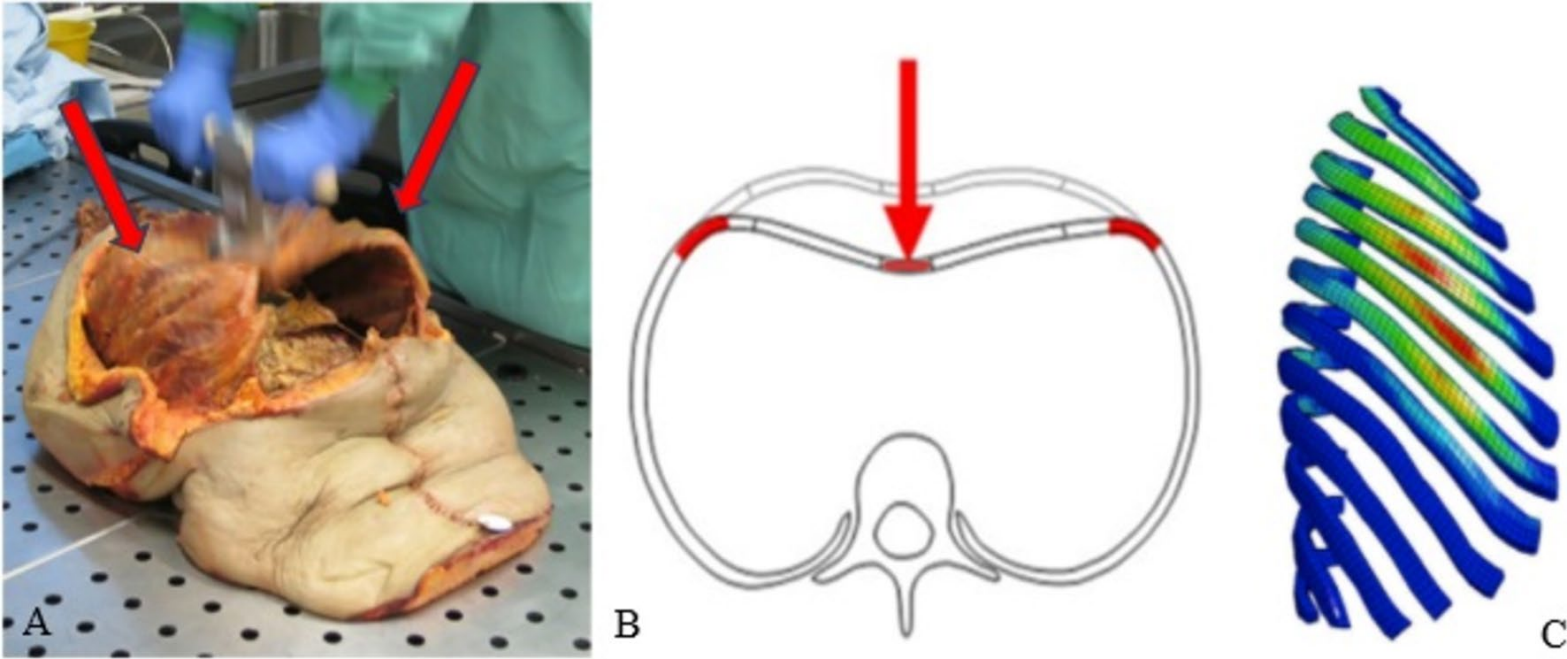
**A** Performing CPR on a torso showing maximum bending of the ribs anterolaterally (arrows). **B** Fracture mechanism causing direct (arrow) and indirect fractures in CPR [2], **C** simulation results of maximum 1st principal strain in the ribs, from force–deflection sensor loading, at 100 compressions per minute, and 5 cm depth. Red: highest values within each rib [16]

We hypothesize that rib fractures occurring during CPR exhibit specific characteristics regarding their morphology, location, type, and degree of displacement. To avoid confounding factors and ensure accurate analysis of the true fracture mechanism, we included only patients who received CPR after a non-traumatic cardiac arrest. CPR-related fractures are chosen for their distinct mechanism: force is applied directly to the sternum, which transmits to the ribs, causing indirect fractures in the lateral shaft area. Biomechanical and finite element models explain these fracture patterns, as the highest tension values are typically reached in the lateral region of the ribs. (Fig. 1).

## Methods

We conducted a retrospective analysis of all inpatients who received CPR following non-traumatic cardiac arrest and were subsequently treated for traumatic chest wall fractures (ICD-10 22.2, 22.3, 22.4, 22.5) at a Level 1 Trauma Centre between 2010 and 2016. Each patient had undergone appropriate computed tomography (CT) of the thorax (n = 40).

Clinical data retrieved from the patients’ medical records included age and sex. Patients were stratified into subpopulations based on fracture location (anterior, lateral, posterior), and all CT scans were analyzed using Magic-Web (Siemens, Munich). CT analysis involved basic anatomical measurements of the thoracic cage to determine thoracic width and depth, and precise measurements of the ribs and sternum (diameter, osteochondral junction position, and angle) were taken in the bony window, with cartilage examined in the soft tissue window.

Primary endpoints included the number, morphology, location, type, and degree of displacement of rib and cartilage fractures. Secondary endpoints were the occurrence of incomplete fractures and their clinical relevance. We also analyzed type B fractures of the rib shaft for characteristic fracture morphologies.

**Fig. 2:**
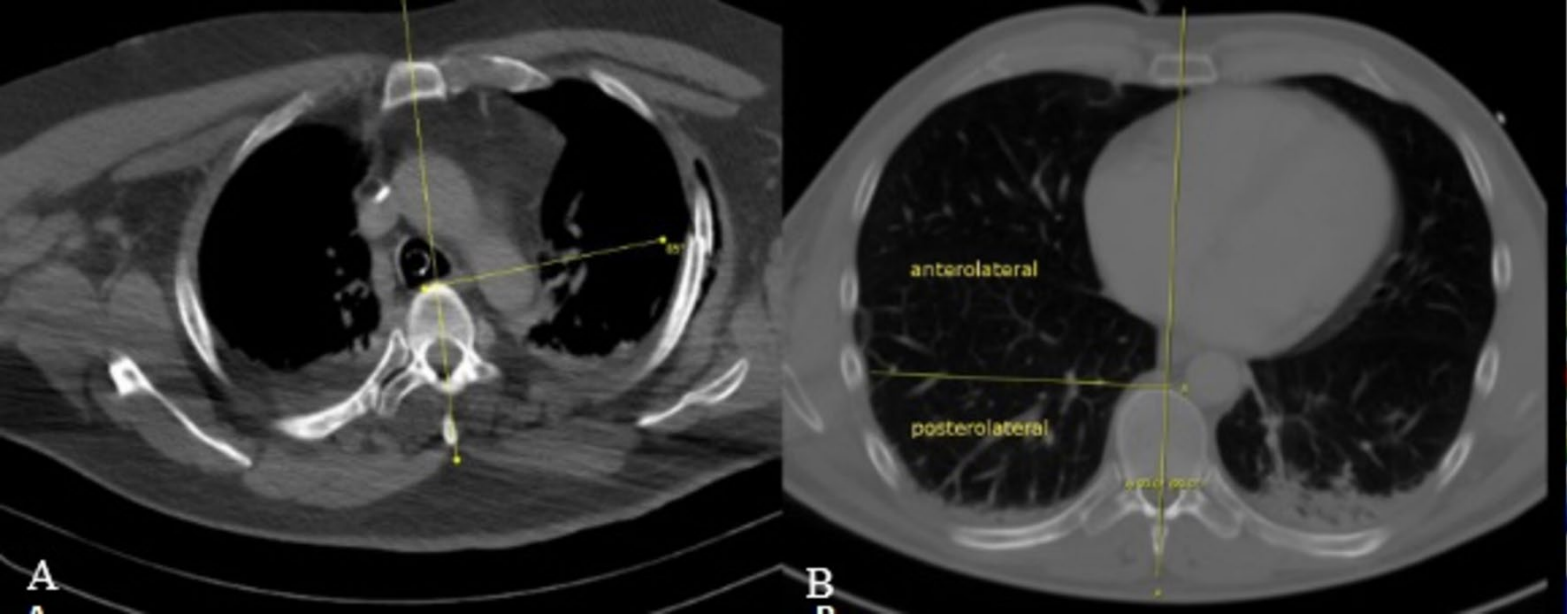
**A** Example of a straight rib fracture at 85° in axial plane. **B** Anterolateral (< 90°) and posterolateral (≥ 90°) sector of the shaft segment

Fracture location was assessed in the axial plane. To determine each fracture’s position, the apex point was centered on the anterior edge of the respective vertebral body, while the fixed-angle leg was extended from this point along a line from the spinous process through the vertebral center to the center of the sternum or, more caudally, the linea alba. The variable angle leg was then placed at the fracture point being measured (Fig. 2).

**Fig. 3:**
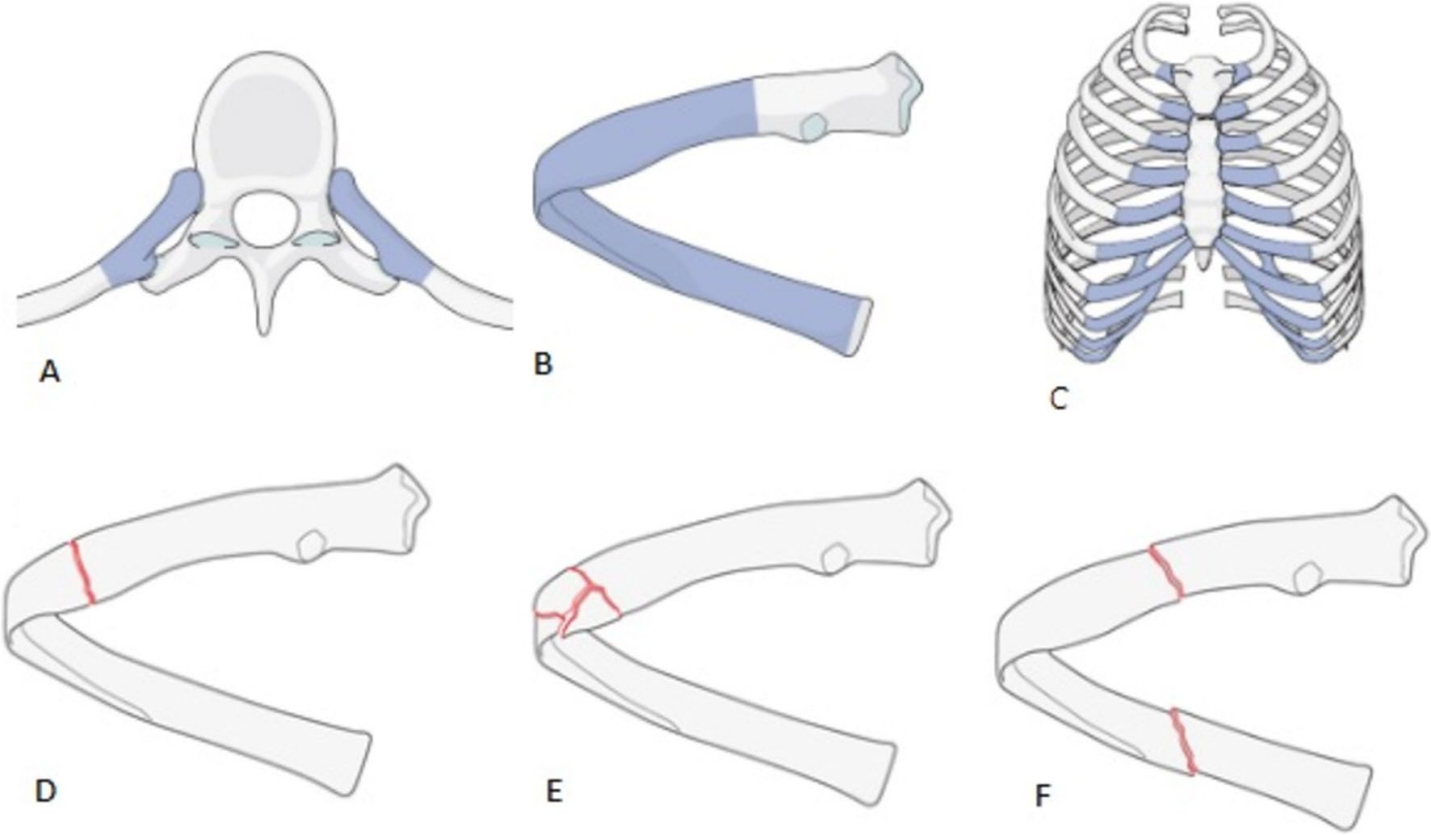
Classification of rib segments and fractures according to AO & OTA [11]: **A** posterior end segment, **B** bone between the two end segments, **C** costochondral cartilage and CCJ, **D** simple type A fracture, **E** multifragmentary type B fracture, **F** multifragmentary segmental type C fracture

To classify fracture position, we utilized the AO/OTA 3-segment model (Fig. 3) [11]: Segment 1, the posterior end segment: extended from the costovertebral joint to the tip of the transverse process (costotransverse articulations), Segment 2, the shaft segment: spanned the bone between the two end segments, and Segment 3, the anterior end segment: the costochondral cartilage (CC) and costochondral joint (CCJ). Within the shaft segment, we divided fractures into anterolateral (< 90°) and posterolateral (≥ 90°) regions (Fig. 2).

**Fig. 4:**
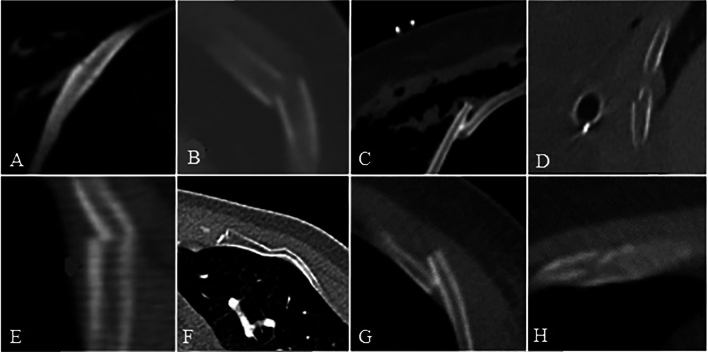
Morphology of rib fractures: **A** undisplaced, **B** offset, **C** displaced, **D** outer cortex affected, **E** inner cortex affected, **F** buckle fracture, **G** straight fracture line, **H** oblique fracture line

Fractures were categorized per the AO/OTA system [11] as simple type A fractures, multifragmentary wedge type B fractures, and multifragmentary segmental type C fractures (a combination of two fractures: AA/AB/BB). Local comminutions were classified as type B fractures. Additionally, incomplete fractures, in which only one cortex was affected, were identified and assessed for potential displacement (e.g., ad axim) (Fig. 4).

Type A fractures were analyzed for fracture line orientation following the approach of Kissling and Hausmann. We defined a straight fracture as one without an offset between internal and external fracture edges and an oblique fracture as one with such an offset. No differentiation was made between ventral and dorsal offsets, as previous findings showed no significance in relation to fracture type.

Type B fractures were further examined for the presence of wedges or comminuted fractures. Each rib classified as type A or B was double-checked for additional fractures within or beyond the same subsegment; in cases of multiple fractures, these were designated as type C fractures (Fig. 3).

Displacement was categorized (undisplaced, offset, displaced) following the Chest Wall Injury Society taxonomy, as updated by the American Society for Emergency Radiology (ASER) [12, 13]: undisplaced ribs with ≥ 90% cross-sectional overlap, offset rib fractures with < 90% overlap, and displaced rib fractures with no cross-sectional overlap (Fig. 4).

Institutional review board approval was obtained, and the Strengthening the Reporting of Observational Studies in Epidemiology (STROBE) guidelines were followed to ensure thorough reporting of our methodology, results, and discussion [14].

## Results

### Descriptive statistics

We included 40 patients who received CPR following non-traumatic cardiac arrest. Among them, 15 were female (37.5%) and 25 male (62.5%), with ages ranging from 40 to 89 years (mean: 69.2 years). As noted, no data was available on the usage of mechanical chest compression devices, which may represent a potential bias.

Across these patients, a total of 424 rib fractures were identified, evenly distributed between both sides of the thorax, with 211 fractures on the right side (mean: 5.3 fractures per patient, range: 0–21) and 213 on the left side (mean: 5.3 fractures per patient, range: 0–13). We found a total of 10.6 fractures per patient.

### Fracture position

**Fig. 5:**
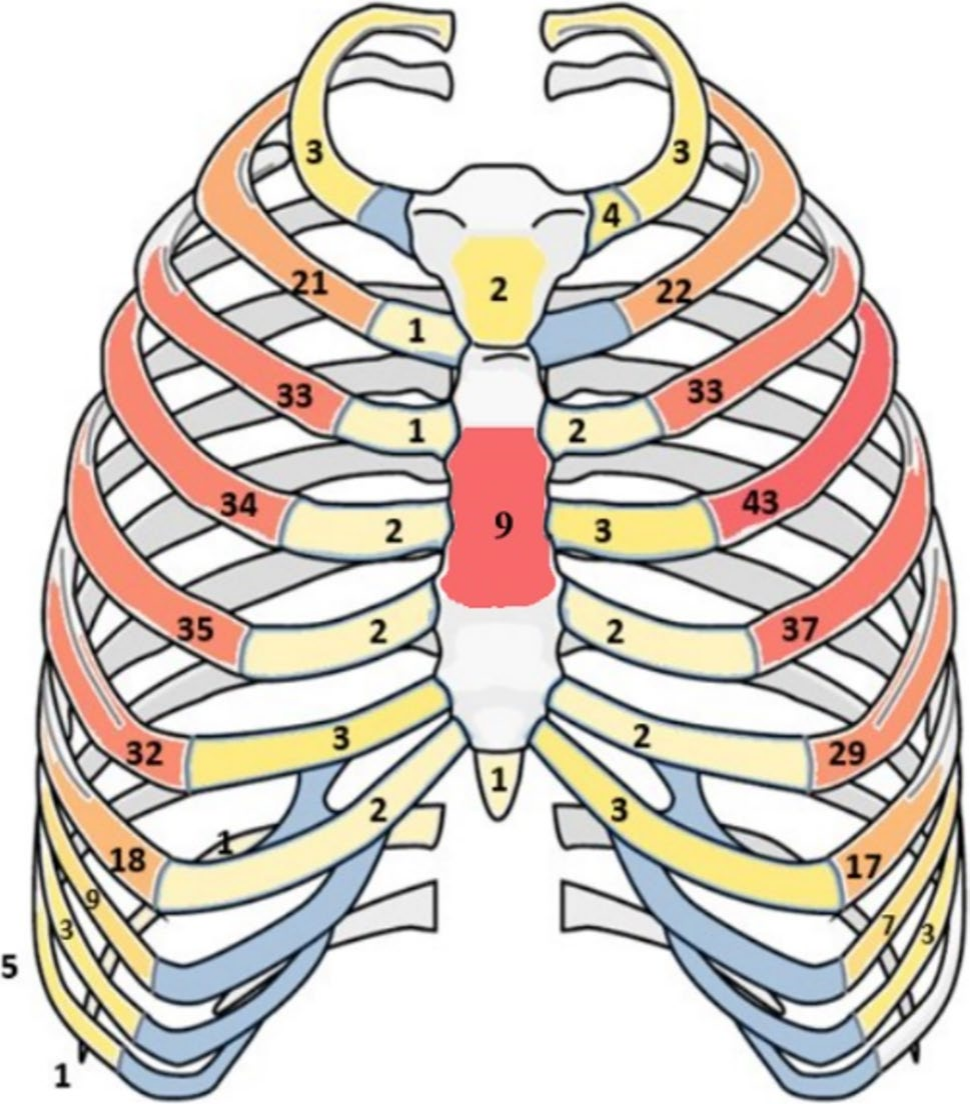
Distribution of rib fractures

Of the fractures observed, 391 (92.2%) were located in the shaft segment, with 371 (94.8%) of these being anterolateral. Additionally, there were 27 fractures (6.4%) involving the costal cartilage (CC) or costochondral junction (CCJ) and 6 (1.4%) posterior fractures (Fig. 5).

### Fracture type

Of the total 424 fractures, 387 (91.3%) were classified as simple type A fractures. There were 37 type B fractures (8.7%), predominantly on the left side (left: 24 [64.9%] vs. right: 13 [35.1%]). Among type B fractures, 97% (n = 36) were local comminutions confined to within one shaft width. We identified one classic wedge fracture among these cases. Ten of the type B fractures (27%) were also part of type C fractures, in which two fractures were observed within or across subsegments.

Type C fractures were more evenly distributed across the thorax, with 30 fractures (52.6%) on the left and 27 (47.4%) on the right, though these differences were not statistically significant. The majority of type C fractures (63%) were combinations of two fractures within the shaft segment. Approximately 26.3% of type C fractures were a combination of shaft and anterior fractures, with the CCJ affected in 53% and cartilage fractured in 47%. In 29.8% of type C fractures, one component was incomplete.

We also observed 143 incomplete fractures. Of these, 18 (12.6%) involved the outer cortex, 49 (34.3%) the inner cortex, and 76 (53.1%) were buckle fractures with kinking of the inner cortex.

### Fracture line orientation

Of the 387 type A fractures, 224 (57.9%) could be assessed for fracture line orientation, excluding incomplete fractures and CT scans that were non-diagnostic (n = 20). Among these, 164 fractures (73.2%) had straight fracture lines, and 60 (26.8%) displayed oblique fracture lines. There was no significant difference in the occurrence of straight versus oblique fracture lines between the left and right sides of the thorax.

### Displacement

Out of 424 fractures, 421 were assessed for displacement. We found that 268 fractures (63.7%) were undisplaced, 104 (24.7%) displayed an offset, and 49 (11.6%) were displaced. Among undisplaced fractures, 53.4% were incomplete. A statistically significant difference was noted in the offset fractures between the right and left sides (38 vs. 66 fractures, p < 0.05).

### Association of fracture line orientation and degree of displacement

In type A fractures with complete fractures, both straight and oblique fracture lines showed approximately equal distribution among undisplaced, offset, and displaced fractures (Table 1).

**Table 1:** Association of fracture line orientation and degree of displacement

Straight n = 164			Oblique n = 60		
Undisplaced	Offset	Displaced	Undisplaced	Offset	Displaced
81 (49.4%)	54 (32.9%)	29 (17.7%)	29 (48.3%)	20 (33.3%)	11 (18.3%)

### Sternum and anterior rib fractures

**Fig. 6:**
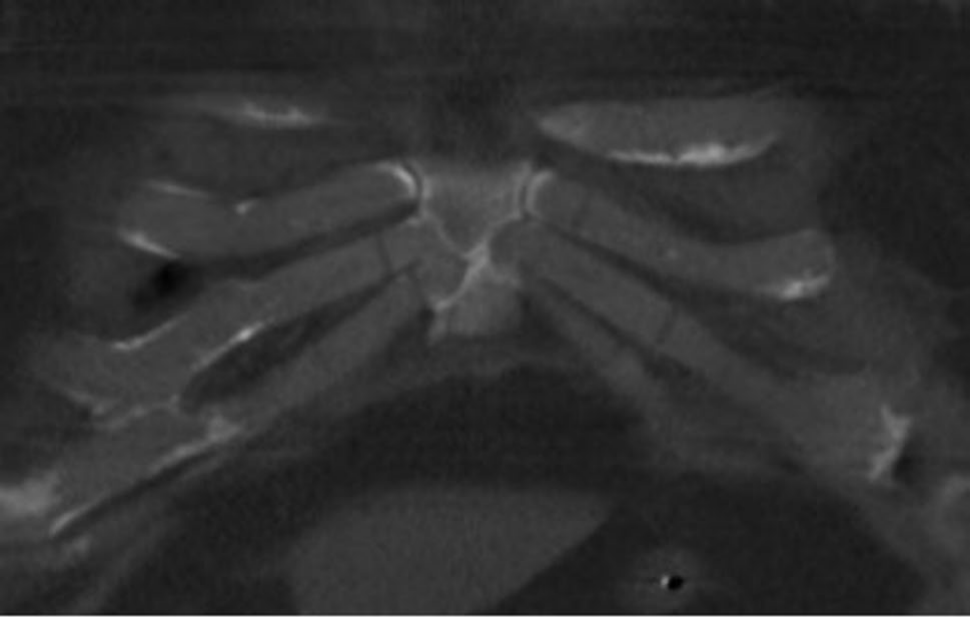
All anterior fractures of the cartilage are straight type A fractures

Sternum fractures were present in 30% (n = 12) of patients, with 50% located at the corpus sterni between ribs 3 and 4. Two-thirds of these were classified as type A fractures following *under *the AO/OTA system. Patients with sternum fractures had significantly more rib fractures (total: 156, mean: 13 fractures per patient, p < 0.01). These patients were generally older (mean age: 71.5 years) and exhibited fractures on the left thorax, with 91.6% also showing fractures on the right. Anterior fractures of the cartilage or osteochondral junction were present in 83% of these patients, all characterized as straight, undisplaced type A fractures (Fig. 6).

## Discussion

The primary objective of this paper was to analyze the morphology, location, type, and degree of displacement of rib fractures sustained during CPR, given that the fracture mechanism is well-documented in both human and animal models, as well as in biomechanical studies [1–5, 15, 16]. Our findings reveal that over 80% of fractures occurred between the 3rd and 7th ribs, symmetrically on both sides of the thorax. These fractures were most frequently located anterolaterally at a 50–60° angle in the axial plane, corresponding to the midclavicular line—a finding that aligns with previous studies and finite element model validations [3, 4].

Approximately one-third of our patients sustained sternum fractures, primarily at the 3rd to 4th intercostal space, where CPR force, whether manual or mechanical, is typically applied. Notably, patients with sternum fractures had, on average, 20% more rib fractures, likely due to the higher forces exerted during resuscitation. This finding is consistent with the increased morbidity and mortality rates observed in severely injured patients who sustain additional sternum fractures (SF) in flail chest injuries [17]. Furthermore, costal cartilage fractures or costochondral tears were present in all patients with sternum fractures, with half of these cartilage fractures part of a type C fracture. Liebsch et al. [2] similarly noted that impact loading likely transfers to adjacent structures rather than affecting isolated structures alone.

Kissling and Hausmann [7] previously observed that “direct breaking mostly produced straight fractures, whereas indirect breaking produced similar proportions of straight and oblique fractures.” However, this finding was based on isolated ribs subjected to slow force application. In our in vivo analysis, we observed approximately 2–3 times as many straight fracture lines as oblique ones, which may be due to the thorax’s complex anatomy and dynamic biomechanics during resuscitation.

A significant portion of the fractures (33%) were incomplete, exhibiting monocortical or buckle features, similar to greenstick fractures in pediatric cases. This underscores the unique properties of rib bones, which retain considerable elasticity even in advanced age. Buckle fractures, which are often visible only in soft tissue window CT images, are typically missed on initial scans [18]. These fractures commonly occur in ribs 2 to 6 laterally and ribs 7 to 9 near the costochondral junction. Yang et al. [6] attributed this to structural features of distal ribs, such as cortical plate flaring to accommodate adjoining cartilage thickness.

Incomplete fractures were frequently components of type C fractures, highlighting the importance of scanning the entire rib once an initial fracture is identified. Over time, incomplete fractures could become complete due to minor traumas such as coughing, sneezing, or laughing. Therefore, clinical and radiological follow-up is advisable. Buckle fractures may also contribute to flail chest segments and should be factored in when planning surgical stabilization. Further research into their clinical impact, including pain persistence or increased pneumonia rates, may be beneficial.

We propose that incomplete rib fractures be considered a distinct category within the spectrum of fracture displacement. Notably, we did not observe wedge fractures typical of long bones. Instead, fractures presented as multifragmentary or exhibited local comminution within a single shaft width, suggesting that a more tailored definition of type B fractures for rib injuries may be appropriate. No type B fractures were identified in the cartilage, a finding consistent with Groh et al. [19].

Klöss et al. [20] have previously distinguished between injury patterns from CPR and accidental trauma. The findings from this study may contribute to forensic and trauma medicine, providing insight into thoracic injury mechanisms and aiding in trauma reconstruction.

We acknowledge limitations in this study, including the potential for fractures to have been missed during initial examinations, especially incomplete fractures that are difficult to detect on initial CT scans or cartilage fractures that may only be visible in the soft tissue window. This may result in underestimating the true extent of injuries. Additionally, no data were available regarding the use of mechanical chest compression devices during CPR. Since the force and pattern of fracture formation may vary between manual and mechanical CPR, the absence of this data represents a potential source of bias. Furthermore, as this was a single-center study, the findings may not be generalizable to other institutions or patient populations with different CPR protocols or resuscitation settings.

Further investigation into different fracture patterns resulting from direct blunt trauma, such as those due to lateral impacts or crush injuries, is warranted. Differentiating these patterns poses a challenge, especially when detailed medical histories and injury mechanisms are unavailable. It is anticipated that lateral impact traumas may exhibit more direct fractures in the lateral segment of the chest wall, with indirect fractures occurring anteriorly or posteriorly, and a higher incidence of type B fractures with local comminution. Detailed analysis of cases involving distinct and reproducible impacts, such as those seen with steering wheel or seatbelt trauma could yield insights into how these injuries differ from CPR-related fractures.

Enhanced understanding of these fracture patterns may also aid in refining injury classification and treatment planning, particularly for incomplete and buckle fractures that may develop into flail segments or complicate recovery. This research could contribute to forensic medicine, enabling more precise trauma reconstructions and possibly informing guidelines for improved rib fracture management.

## Conclusion

In this study, we characterized the morphology, location, type, and displacement of rib fractures resulting from CPR-related trauma. Our findings highlight unique fracture patterns, with a high prevalence of incomplete and monocortical fractures, which suggests rib elasticity persists even with age. Identifying fracture patterns specific to CPR may improve clinical assessment, especially in the context of delayed or undetected fractures. Further studies on fracture morphology from diverse trauma mechanisms could enhance forensic and clinical insights, ultimately improving patient outcomes and treatment strategies.

## Data Availability

Data is provided on request by the publisher.
